# Impact of a prognostic model for overall survival on the decision‐making process in a head and neck cancer multidisciplinary consultation meeting

**DOI:** 10.1002/hed.27163

**Published:** 2022-07-30

**Authors:** Maarten C. Dorr, Arta Hoesseini, Aniel Sewnaik, José A. Hardillo, Robert J. Baatenburg de Jong, Marinella P. J. Offerman

**Affiliations:** ^1^ Department of Otorhinolaryngology and Head and Neck Surgery, Erasmus MC Cancer Institute Erasmus University Medical Center Rotterdam The Netherlands

**Keywords:** head and neck cancer, multidisciplinary decision making, prognostic counseling, shared decision making, mixed method study

## Abstract

**Background:**

Multidisciplinary decision‐making in head and neck cancer care is complex and requires a tradeoff between prolonging survival and optimizing quality of life. To support prognostication and decision‐making in head and neck cancer care, an individualized prognostic model for overall survival (OncologIQ) is available.

**Methods:**

By quantitative and qualitative research we have studied user value of OncologIQ and its impact on the decision‐making process in a multidisciplinary consultation meeting.

**Results:**

Healthcare professionals experienced added value upon using prognostic estimates of survival from OncologIQ in half (47.5%) of the measurements. Significant impact on the decision making process was seen when OncologIQ was used for older patients, patients having a WHO performance score ≥ 2, or high tumor stage.

**Conclusions:**

The prognostic model OncologIQ enables patient‐centered decision‐making in a multidisciplinary consultation meeting and was mostly valued in complex patients.

## 1 INTRODUCTION

Decision‐making in head and neck cancer care requires a tradeoff between prolonging survival and optimizing quality of life (QoL). The multidisciplinary consultation meeting (MCM) is therefore pivotal in the oncological workup. The MCM ensures that tumors are accurately staged, and treatment plans are evidence‐based and reached by consensus.^1^, ^2^, ^3^, ^4^ However, making well‐informed and patient‐centered treatment plans remains challenging.^5^, ^6^, ^7^, ^8^, ^9^, ^10^, ^11^, ^12^, ^13^ All patient and tumor‐related variables should be available and considered structurally by the MCM.^5^, ^6^, ^7^ Weighing all these variables is complex, and healthcare professionals may face difficulty in making accurate individual survival predictions.^14^, ^15^

To support prognostication and decision‐making in head and neck cancer (HNC), an internally and externally validated prognostic model named OncologIQ has been developed by the head and neck department of the Erasmus MC. This model estimates the 1‐ to 10‐year overall survival (OS) chances of patients with primary HNC, based on the average treatment effect.^16^, ^17^, ^18^, ^19^, ^20^, ^21^, ^22^ Apart from tumor data, it includes other patient‐specific factors, such as age, comorbidity, performance status, and socioeconomic status. Prognostic models are increasingly developed and it is advocated that prognostic models could support and individualize the decision‐making process, for example, during MCMs and doctor‐patient consultations. However, more research is necessary for evaluating the impact in clinical practice.^23^, ^24^, ^25^

The overall aim of this study was to explore user value of the prognostic model OncologIQ and its impact on the decision‐making process in a head and neck cancer multidisciplinary consultation meeting. This was done by measuring: (1) perceived added value of the use of OncologIQ; (2) therapeutic doubt in the multidisciplinary treatment plan; and (3) adjustments in the multidisciplinary treatment plan due to OncologIQ. User value was assessed by qualitative interviews with healthcare professionals from the MCM.

## 2 MATERIALS AND METHODS

We conducted a mixed method study to explore user value and impact of the prognostic model OncologIQ in the Erasmus MC head and neck cancer MCM. For this study, the explanatory design was used. This comprises qualitative data collection during a second phase as follow‐up to the quantitative data. This design enables us to use qualitative outcome data to better understand quantitative outcomes.^26^

### 2.1 OncologIQ

**FIGURE 1:**
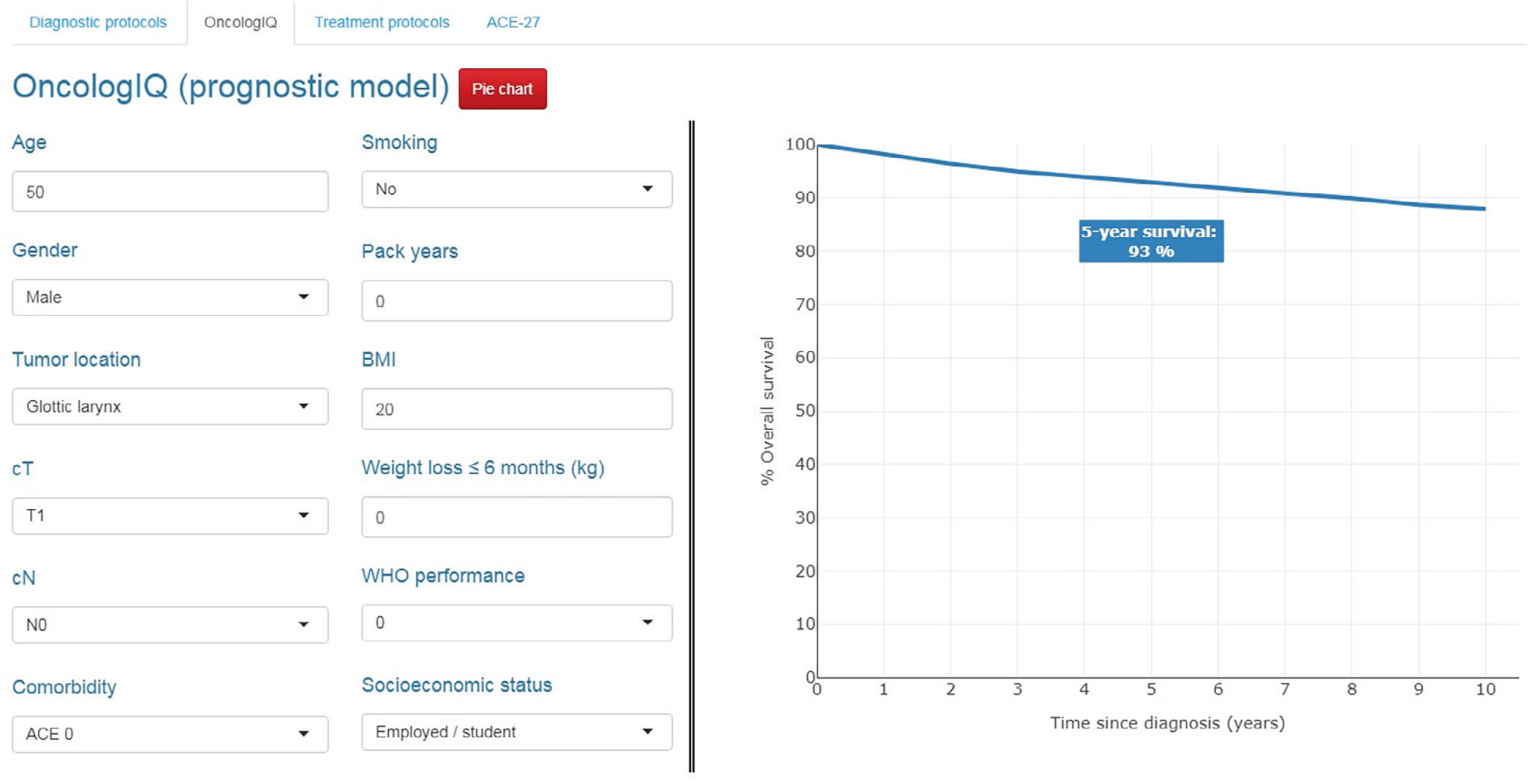
An example of OncologIQ, as used in the multidisciplinary disciplinary team [Color figure can be viewed at wileyonlinelibrary.com]

OncologIQ is an internally and externally validated prognostic model which supports shared decision‐making for patients with primary HNC.^16^, ^17^, ^18^, ^19^, ^20^, ^21^, ^22^ This model estimates the 1‐ to 10‐year overall survival chances (OS) of patients with primary HNC, based on the average treatment effect. It combines TNM‐classification with the following patient‐specific predictors: age, sex, comorbidity, tumor location, smoking, BMI, weight loss, WHO performance, and socioeconomic status. OncologIQ includes the following tumor locations: lip, oral cavity, oropharynx, nasopharynx, hypopharynx, and larynx. The current model is developed for patients with a primary curative tumor and does not apply to secondary primary tumors, recurrent or noncurative disease. The model can be found at www.oncologIQ.com. An example can be seen in Figure 1.

### 2.2 Institutional routine

All newly diagnosed patients from the department of head and neck surgery and oral and maxillofacial surgery of the Erasmus MC were discussed during the weekly MCM. The attending medical specialties were head and neck surgery, radiation oncology, oral and maxillofacial surgery, medical oncology, radiology, and geriatrics. Patients were presented by their own treating specialist and discussed according to local and national guidelines.^1^, ^27^

### 2.3 Setting and participants

Six meetings were attended by the research team. The decision for six meetings was made in agreement with the healthcare professionals, based on feasibility for this study, and to avoid bias due to a learning effect after more meetings. All healthcare professionals involved in the decision‐making participated in this study. Patients were included if diagnosed with a primary head and neck squamous cell carcinoma (HNSCC) of the lip, oral cavity, larynx, oropharynx, nasopharynx, or hypopharynx, and eligible for curative treatment. Exclusion criteria were synchronous primary or recurrent HNSCC. This study is part of a prospective cohort study, which was approved by the ethics committee of the Erasmus MC (MEC number: MEC‐2013‐052). All participants provided written informed consent.

### 2.4 Research team

The research team consisted of three main investigators. M.P.J. Offerman (MO), PhD and psychologist; A. Hoesseini (AH), MD, PhD‐candidate, and clinical epidemiologist; M. Dorr (MD), MD and PhD‐candidate. Both MO and AH have experience with conducting qualitative research.^19^, ^28^, ^29^ The researchers were not members of the MCM. A work relationship exists between the participating healthcare professionals and the research team. MO and AH are co‐developers of the prognostic model OncologIQ.

## 3 QUANTITATIVE RESEARCH

### 3.1 Main outcomes and design

During the MCM a six‐step design was used. Main outcomes and measures were:

1. Perceived added value of the use of OncologIQ.

2. Therapeutic doubt in the multidisciplinary treatment plan.

3. Adjustments in the multidisciplinary treatment plan with respect to the use of OncologIQ.

Patients were discussed in the MCM according to the standard way of working *(step 1)*. After formulating a treatment plan for the individual patient, all healthcare professionals were asked to rate their individual *therapeutic doubt* in making a well‐founded multidisciplinary treatment plan with the available information “as normal” on a 10‐point visual analogue scale (VAS) scale for this specific patient *(step 2)*. Thereafter, the personalized prognostic information from OncologIQ was displayed on a screen *(step 3)*. Again, the professionals rated their *therapeutic doubt* on a 10‐point VAS scale *(step 4)* for this specific patient. The healthcare professionals were asked if they would reconsider the treatment plan given the supplementary prognostic information *(step 5)*. The research team (MD, AH) noted any adjustments. Finally, the professionals scored their perceived *added value* of the use OncologIQ for the specific patient on a 4‐point Likert scale *(step 6)*. These steps were repeated for every patient.

### 3.2 Analyses

Statistical analyses were performed using SPSS version 25.0.^30^ There were no missing data. Descriptive statistics were used to calculate frequencies and proportions. Added value was scored on a 4‐point Likert scale, but converted to a binomial variable for further analyses. For therapeutic doubt, a delta value was calculated and categorized as more doubt, less doubt, or no change. These are used as categorical data for further analysis. For categorical data, the Pearson Chi‐squared test and Fisher's exact test were used when appropriate to assess heterogeneity between groups. For continuous data, the Student's *t* test and the analysis of variance (ANOVA) model were used. Statistical significance was established at *p* < 0.05.

## 4 QUALITATIVE RESEARCH

### 4.1 Main outcomes and design

After six MCMs, structured interviews with the healthcare professionals were conducted. The interviews were held by a male researcher (MD). Questions were prepared via a structured interview guide and discussed previously by the research team (MD, AH, MO). The healthcare professionals were asked about whether they did or did not experienced the use of OncologIQ as added value. Questions from the interviews can be found in Appendix A. All participating healthcare professionals who attended at least two MCMs were approached by email to participate in these structured interviews after finishing the quantitative study. A minimum of two meetings attended was chosen because experience with the use of OncologIQ within the MCM is needed. The interviews were held at the hospital and took 20 min each. Interviews were not repeated. The interviews were audio recorded and transcribed (MD) in Microsoft Excel. No field notes were available. As part of the interviews, suggestions for future use were explored. In addition, the Net Promoter Score was measured. This score measures the likelihood for recommending OncologIQ to other colleagues. This is measured on a Likert scale from 0 (do not recommend) to 10 (will definitely recommend).

### 4.2 Analyses

The theoretical framework of phenomenology was used to analyze the data and determine healthcare professionals experience with OncologIQ. Three researchers (MD, AH, MO) coded all transcripts. After individual analysis of the data, inductive categories were derived during three intensive sessions. Consensus was reached by discussion. When a given answer needed more elaboration, the healthcare professional was asked for more details. Participants did not provide feedback on the findings. As all available healthcare professionals were interviewed, we did not consider data saturation. Qualitative results are described using the consolidated criteria for reporting qualitative research (COREQ).^31^

## 5 RESULTS

### 5.1 Quantitative results

In six MCMs, the supplementary prognostic information for 38 patients was included during the decision‐making process. A total of 419 measurements were retrieved from 18 healthcare professionals. Participating healthcare professionals consisted of seven head and neck surgeons, five radiation oncologists, two medical oncologists, two physician assistants, one otorhinolaryngology, and one maxillofacial surgery resident. Not every healthcare professional attended every meeting. Baseline patient characteristics are summarized in Table 1.

**TABLE 1:** Baseline characteristics

No. of patients	38
No. of measurements	419
Mean age, years (SD)	65.6 (11.4)
Sex	
Men	30 (78.9%)
Women	8 (21.1%)
ACE‐27	
0 (none)	13 (34.2%)
1 (mild)	15 (39.5%)
2 (moderate)	6 (15.8%)
3 (severe)	4 (10.5%)
WHO	
0	29 (76.3%)
1	4 (10.5%)
2	4 (10.5%)
3	1 (2.6%)
Smoking	
No	3 (10.5%)
Yes	21 (55.3%)
Former	13 (34.2%)
Mean PY (SD)	26.0 (15.7)
Mean weight loss (SD)	1.3 (2.4)
Mean BMI (SD)	24.2 (4.2)
Employment	
Retired	22 (57.9%)
Yes	11 (28.9%)
No	5 (13.2%)
Tumor localization	
Larynx	9 (23.7%)
Oral cavity	12 (31.6%)
Oropharynx	11 (28.9%)
HPV‐positive	2 (18.2%)
HPV‐negative	9 (81.8%)
Hypopharynx	6 (15.8%)
Tumor stage	
I	12 (31.6%)
II	4 (10.5%)
III	8 (21.1%)
IV	14 (36.8%)
Treatment plan	
Surgery	9 (23.7%)
Radiotherapy	11 (28.9%)
Surgery AND radiotherapy	6 (15.8%)
Surgery OR radiotherapy	4 (10.5%)
Chemo radiation	7 (18.4%)
Curative OR palliative radiotherapy	1 (2.6%)

### 5.2 Added value

Table 2 displays the added value according to the healthcare professionals. In nearly half (47.5%) of the measurements, the healthcare professionals experienced added value in using OncologIQ during the MCM: 125 times (29.8%) as low added value, 71 times (16.9%) as moderate and, three times (0.7%) as high added value. Patients for whom the prognostic information was considered to be of added value were significantly older (*p* = 0.02), had a WHO performance score of ≥ 2 (*p* = 0.001), and tumor stage IV (*p* ≤ 0.001). The median 2‐ and 5‐year survival chances were significantly lower in the added value group (*p* < 0.001).

**TABLE 2:** Added value of OncologIQ score according to healthcare providers

	No added value	Added value	Sig*
No. of measurements	220 (52.5%)	199 (47.5%)	
Mean age, years (SD)	64.6 (10.6)	67.1 (11.3)	*0.02*
Sex			
Men	179 (81.4%)	151 (75.9%)	0.17
Women	41 (18.6%)	48 (24.1%)	
ACE‐27			
0	78 (35.5%)	59 (29.6%)	0.24
1	91 (41.4%)	78 (39.2%)	
2	33 (15.0%)	36 (18.1%)	
3	18 (8.2%)	26 (13.1%)	
WHO			
0	177 (80.5%)	138 (69.3%)	*0.009*
1	23 (10.4%)	23 (11.6%)	
≥2	20 (9.1%)	38 (19.1%)	
Smoking			
No	19 (8.6%)	24 (12.1%)	0.16
Yes	118 (53.6%)	116 (58.3%)	
Former	83 (37.7%)	59 (29.6%)	
Mean PY (SD)	24.9 (16.3)	23.1 (17.1)	0.39
Mean weight loss, kg (SD)	1.4 (2.3)	1.5 (2.4)	0.64
Mean BMI (SD)	24.4 (4.3)	25.0 (5.1)	0.13
Employment			
Retired	115 (52.3%)	131 (65.8%)	*0.007*
Yes	77 (35.0%)	43 (21.6%)	
No	28 (12.7%)	25 (12.6%)	
Tumor stage			
I	96 (43.6%)	40 (20.1%)	*<0.001*
II	11 (5.0%)	30 (15.1%)	
III	45 (20.5%)	44 (22.1%)	
IV	68 (30.9%)	85 (42.7%)	
2‐year median survival (Q1–Q3)	86.0% (72.0–90.0)	73.0% (56.0–86.0)	*<0.001*
5‐year median survival (Q1–Q3)	73.0% (51.0–80.0)	53.0% (31.0–73.0)	*<0.001*

*Note*: *Significance based on residuals.

### 5.3 Therapeutic doubt

Mean therapeutic doubt in the multidisciplinary treatment plan before and after seeing OncologIQ was 1.0 (±1.5) and 1.1 (±1.7) in the total group, respectively. Table 3 displays the change in therapeutic doubt after seeing OncologIQ's estimates of the individuals' survival chances. In 100 (23.8%) measurements, the personalized prognostic information caused a change in therapeutic doubt. In 47 (11.2%) measurements, healthcare professionals expressed less doubt with a mean delta of 1 (±1), and in 53 (12.6%) measurements, they expressed more doubt with a mean delta of 3 (±2) related to the initial treatment plan. Patients for whom the prognostic information caused more therapeutic doubt were significantly older (*p* < 0.001), had moderate or severe comorbidity (*p* = 0.03), a WHO performance score of ≥ 2 (*p* < 0.001), and tumor stage IV (*p* < 0.001). Less therapeutic doubt was experienced regarding patients who were significantly younger (*p* < 0.001), had no or less comorbidity (*p* = 0.003), and with low WHO performance status (*p* < 0.001). Estimated median survival chances differed significantly between the groups (*p* < 0.001).

**TABLE 3:** Change in therapeutic doubt after using OncologIQ

	Less doubt	No change	More doubt	Sig*
No. of measurements	47 (11.2%)	319 (76.1%)	53 (12.6%)	
Mean age (SD)	66.2 (10.8)	64.6 (11.1)	72.8 (6.8)	*<0.001*
Sex				
Men	37 (78.7%)	256 (80.3%)	37 (69.8%)	0.23
Women	10 (21.3%)	63 (19.7%)	16 (30.2%)	
ACE‐27				
0	13 (27.7%)	110 (34.5%)	14 (26.4%)	*0.03*
1	25 (53.2%)	129 (40.4%)	15 (28.3%)	
2	4 (8.5%)	50 (15.7%)	15 (28.3%)	
3	5 (10.6%)	30 (9.4%)	9 (17.0%)	
WHO				
0	40 (85.1%)	252 (79.0%)	23 (43.4%)	*<0.001*
1	6 (12.8%)	37 (11.6%)	3 (5.7%)	
2 + 3	1 (2.1%)	30 (9.4%)	27 (50.9%)	
Smoking				
No	7 (14.9%)	34 (10.7%)	2 (3.8%)	0.25
Yes	27 (57.4%)	172 (53.9%)	35 (66.0%)	
Former	13 (27.7%)	113 (35.4%)	16 (30.2%)	
Mean PY (SD)	23.7 (17.5)	23.9 (16.7)	25.8 (16.1)	0.74
Mean weight loss, kg (SD)	1.7 (2.4)	1.5 (2.4)	0.9 (1.6)	0.16
Mean BMI (SD)	24.9 (5.3)	24.6 (2.7)	24.9 (4.3)	0.84
Employment				
Retired	29 (61.7%)	168 (52.7%)	49 (92.5%)	*<0.001*
Yes	11 (23.4%)	107 (33.5%)	2 (3.8%)	
No	7 (14.9%)	44 (13.8%)	2 (3.8%)	
Tumor stage				
I	14 (29.8%)	119 (37.3%)	3 (5.7%)	*<0.001*
II	1 (2.1%)	24 (7.5%)	16 (30.2%)	
III	16 (34.0%)	65 (20.4%)	8 (15.1%)	
IV	16 (34.0%)	111 (34.8%)	26 (49.1%)	
2‐year median survival (Q1–Q3)	76.0% (72.0–86.0)	83.0% (72.0–90.0)	47.0% (44.0–58.0)	*<0.001*
5‐year median survival (Q1–Q3)	58.0% (52.0–74.0)	68.0% (51.0–80.0)	22.0% (19.0–33.0)	*<0.001*

*Note*: *Significance based on residuals.

### 5.4 Change of multidisciplinary treatment plan

For one patient, the supplementary individual prognostic information led to an adjustment in the treatment plan. Before displaying the estimated survival chances from OncologIQ, there was a consensus for curative treatment with radiotherapy. The displayed 5‐year overall survival chance of 31% led to a discussion about the treatment plan. The multidisciplinary team decided that both curative and palliative radiotherapy should be discussed with the patient. For this patient, the prognostic information provided by OncologIQ was valued moderate to high.

### 5.5 Qualitative results

A total of 15 healthcare professionals participated in the structured interviews about the use of OncologIQ in the MCM. Participants included seven professionals from the head and neck department, five from radiation oncology, two from oral and maxillofacial surgery, and one medical oncologist. One healthcare professional did not participate because he worked elsewhere during the interviews and two only joined the MCM once.

#### 5.5.1 User value

From the structured interviews, we derived six themes: complex patients, patient‐centered care, holistic awareness, individual patient counseling, protocol‐based care and concerns. These main themes are divided in “added value” or “no added value”, which is in line with the overall construct of our study and research question. These themes and verbatim examples can be found in Table 4.

**TABLE 4:** (Sub)themes and quotations, derived from the structured interviews exploring user value

	Theme	Quotation
Added value	Complex patients: OncologIQ provides useful predictions when the MCM is confronted with therapeutic dilemmas in patients with advanced tumors, higher age, more comorbidity and higher WHO performance score.	“The information is of added value for complex patients where extensive treatment is the only option and doubt about curative intention could arise” (3.14) “In complex patients, where the prognosis is important but more difficult to predict due to age and comorbidity” (3.2) “When there is a poor 2‐year prognosis, there is more substantiation for waiving impactful treatment” (2.12)
	Patient centered care: The information from OncologIQ enables a more tailor‐made approach in the decision making process.	“OncologIQ ensures more individualized patient care” (2.2) “Protocols are often used under the guise ‘we always do it this way’, exact outcomes provide a more tailored approach” (2.10)
	Holistic awareness: Understanding of the individual prognosis and the underlying factors enables a more realistic view of the patients health status.	“It enables a better view on the health status of the patient. Normally, we only look at the tumor and the protocol without incorporating other important patient factors in the decision making” (2.15) “It provides short‐ and long‐term predictions and could therefore support the decision making process” (2.13) “It provides a different view on the prognosis, which could be taken into account by the MCM.” (2.5)
	Individual patient counseling: Awareness of the predicted prognosis is useful at the outpatient clinic in supporting patient counseling.	“Awareness of the predicted outcome could influence patient counseling. In patients where there is a low prognosis, more emphasis could be given on waiving treatment” (2.3) “Due to different views of doctors within the MCM, I think OncologIQ will be of most value in patient counseling. Within the MCM, the more aggressive doctors' opinion—we have to try everything—conflicts with the opinion of the more conservative doctor—not everything that is possible should be done. The outcome from the MCM will be that both options should be discussed with the patient” (2.6 & 3.6)
No added value	Protocol‐based care: OncologIQ is less informative for patients with straightforward protocol based treatment.	“For patients with curable disease that fit protocol well, OncologIQ will be less contributive to the decision making process” (2.16)
	Concerns: Concerns could arise about the consequences of using OncologIQ in situations where it is used for waiving treatment.	“Based on a bad predicted prognosis, sometimes it could be dangerous to decide for no curative treatment intention” (2.7) “It could be confusing when we want to treat the patient, but there is a poor predicted prognosis and no other treatment options” (2.8)

#### 5.5.2 Feedback for further use

Suggestions for future use included the integration of the prognostic information into the standard application form used by the multidisciplinary tumor board. Furthermore, these suggestions included the addition of parameters such as prediction of disease‐free survival, quality of life, and toxicity.

#### 5.5.3 Net promoter score

Healthcare professionals would recommend OncologIQ to other healthcare providers on a Likert scale from 0 to 10, with an average of 7.6.

## 6 DISCUSSION

Our overall aim was to explore user value of the prognostic model OncologIQ and its impact on the decision‐making process in a head and neck cancer multidisciplinary consultation meeting (MCM).

Our quantitative results showed that healthcare professionals experienced added value in the use of OncologIQ within the MCM in nearly half (47.5%) of the measurements. This was associated with a higher age of patients, high WHO performance status, higher tumor stage, and therefore lower estimated survival chances. No added value was associated with lower age, low WHO performance status, tumor stage I, and therefore higher estimated survival chances. Our qualitative results are in line with these results: healthcare professionals mentioned to value OncologIQ most in complex patients when confronted with therapeutic dilemmas. Patients were considered complex when they were older, had advanced tumors, more comorbidity, or higher WHO performance score. Other themes that showed the added value of OncologIQ were the ability to improve patient‐centered care, holistic awareness and provide the foundation on which patient and treating healthcare professional are able to make a well‐informed and shared decision. Previous elaboration on the development and benefit of prediction models for clinical practice are in line with our qualitative results.^25^

By measuring therapeutic doubt before and after the use of OncologIQ, we tried to quantify the extent to which healthcare professionals would feel less or more doubtful about making a well‐founded multidisciplinary treatment plan after receiving supplementary prognostic information. Overall therapeutic doubt was low, which we believe can be attributed to our protocolled approach.^12^, ^32^ This is also mentioned in our qualitative outcome. Surprisingly, we found that the cases in which healthcare professionals experience more or less therapeutic doubt after the use of OncologIQ were equally distributed. Moreover, more and less doubt was associated with respectively a lower and higher estimated median survival chance. We would argue that both the experience of less and more doubt would impact the decision making process. A good—maybe expected—prognosis could empower the MCM in their decision‐making and decreases doubt. On the other hand, a low prognosis—maybe unexpected—could increase doubt. This would suggest the patient is more complex and it would create more awareness regarding the underlying prognostic factors. This phenomenon corresponds with the qualitatively obtained theme “holistic awareness”, which mentions the realistic view of a patients' health status by understanding the individual prognosis from OncologIQ.

In our study, the estimated prognosis led to a change in the multidisciplinary treatment plan once. Consequently, the use of OncologIQ was valued by all healthcare professionals in this specific patient.

### 6.1 Strengths and limitations

This study can be considered unique, as this evaluation step is often left out in prognostic research. The results of this study can guide further implementation of OncologIQ in clinical practices. A major strength of this study was the use of the prognostic model OncologIQ, which has been internally and externally validated.^16^, ^17^, ^18^, ^19^, ^20^, ^21^, ^22^ This prognostic model is a practical web‐based tool that is easily accessible during the MCM. The current model is however only developed for patients with a primary curative tumor and does not apply to secondary primary tumors, recurrent or noncurative disease. Other strengths were the participation of many healthcare professionals every meeting and the obtained qualitative data on user value during interviews with the healthcare professionals. A limitation can be found in the fact that this was a single‐center study and it is unclear whether our conclusions can be generalized to other oncological centers as well. Another limitation is that we were not able to investigate the effect of human papillomavirus (HPV) status on therapeutic doubt and added value due to a small number of HPV positive tumors. We do however acknowledge the possible impact of HPV status and corresponding prognosis on the multidisciplinary decision making, especially when more evidence is available for de‐escalation therapies.^33^ Furthermore, we believe our outcomes can be susceptible for confirmation bias which can be a reason for the little amount of change in treatment plan.

### 6.2 Future perspectives

There is an ongoing paradigm shift in the field of medical decision making and the use of prognostic models. There is an increase in the development of prognostic models, which is accelerated by improved techniques and algorithms for analyzing more complex and larger datasets. However the use of prognostic models in clinical practice is still limited. It is considered important that models are validated and clinically tested.^25^ For OncologIQ, consecutive steps have been taken towards developing a valued and clinically useful prognostic model that is tailored to patients' and physicians' needs.^19^, ^29^ This study is the first step in the implementation of OncologIQ in clinical HNC practice. A current trial with sequential cohorts in the Erasmus MC evaluates the impact of the individualized prognosis from the model OncologIQ during the treatment decision consultations. Currently, a prognostic model for palliative HNC patients is being developed. As suggested by the healthcare professionals and patients as well, including QoL in prediction models would benefit the decision‐making process.^19^ This will be a future objective for our department.

## 7 CONCLUSION

This study showed that in the case of complex patients, healthcare professionals find estimates of survival chances from the prognostic model OncologIQ of added value during the multidisciplinary decision making process. OncologIQ improves patient‐centered care and provides healthcare professionals with a more realistic view on the patients' prospects in term of survival chances. OncologIQ is ready for use as standard of care in multidisciplinary decision‐making.

## FUNDING INFORMATION

This research partly received funding from the National Health Care Institute (Zorginstituut Nederland).

## CONFLICT OF INTEREST

The authors declare no conflict of interest

## Data Availability

Data can be obtained on request. Requests should be directed toward the data management team of the Head and Neck department of the Erasmus MC Cancer Institute (hoofdhalschirurgie@erasmusmc.nl), which has a protocol for approving data requests. Because of restrictions based on privacy regulations and informed consent of the participants, data cannot be made freely available in a public repository.

## APPENDIX A Structured interviews

1. What is your personal experience with OncologIQ?

2. Why is or isn't the personalized prognostic information of added value in the decision‐making process?

3. In case you find OncologIQ of added value in the decision making process, for which patients is this the case?

4. Have you changed your opinion about the use of OncologIQ during the pilot study?

5. Do you see areas of improvement for the use of OncologIQ within the MCM?

6. To what degree would you recommend the use of OncologIQ to other colleagues? (rating from 0 = not recommended at all. 10 = absolutely recommended).
